# Cement-Matrix Composites Using CFRP Waste: A Circular Economy Perspective Using Industrial Symbiosis

**DOI:** 10.3390/ma14061484

**Published:** 2021-03-18

**Authors:** Pierluca Vitale, Rosanna Napolitano, Francesco Colella, Costantino Menna, Domenico Asprone

**Affiliations:** Department of Structures for Engineering and Architecture, University of Naples Federico II-Via Claudio, 21, 80125 Naples, Italy; pierluca.vitale@unina.it (P.V.); rosanna.napolitano@unina.it (R.N.); francesco.colella@unina.it (F.C.); d.asprone@unina.it (D.A.)

**Keywords:** structural behavior, life cycle assessment (LCA), processing, industrial symbiosis, carbon fibers, recycle, cement

## Abstract

This study aims to provide a mitigation strategy for reducing the economic and environmental impacts of carbon fiber wastes deriving from automotive industry. Recycling and reuse in the construction industry is proposed, according to an industrial symbiosis within a circular economy perspective. Specifically, the process consists of repurposing carbon fiber reinforced polymer (CFRP) scraps/waste into new cement-matrix composites, for which the resulting benefits, in terms of mechanical and environmental performance, are herein described. An experimental campaign, starting with a specific heat treatment of CFRP sheets and an accurate dimensional distribution analysis of the short carbon fibers, is presented. The influence of the fiber content and length on both the workability and the mechanical performance of cement-based carbon fiber reinforced mortars is also evaluated. A reduced amount of either sand or cement (up to 8% and 12.8% in volume, respectively) is also considered in the mix design of the fiber reinforced mortars and derives from the substitution of the sand or binder with an equivalent volume of CFRP fibers. The results show a satisfactory increase in compressive and flexural strength in the range 10–18% for the samples characterized by a volume fraction of fibers of approximately 4% and having a 2–5 mm length. Finally, a life cycle assessment (LCA, 14040/14044) was carried out to quantify the environmental burden reductions associated with the implementation of the proposed symbiotic scheme.

## 1. Introduction

The construction industry contributes to the socioeconomic growth of a country, improving the quality of life of its citizens. Only considering the top 100 listed construction companies in terms of revenue, the construction sector reached 1,098,569 Million EUR in 2017 [1]. However, construction-related activities are pivotal also in terms of environmental burdens. For example, on a global scale, around 39% of GHG (greenhouse gas) emissions and approximately 40% of primary energy used results from this sector; in Europe 30% of the waste generated comes from construction and demolition activities [2]. In this sector, cement production embodies a central role, representing 8% of global CO_2_ emissions [3]. For this reason, there is a need to improve construction processes, and especially cement production systems, in order to open the doors towards sustainable practices.

In the last few decades, the construction industry has revealed also an increasing use of carbon fiber reinforced polymers (CFRPs) for applications in new or existing structures. The use of these products is growing rapidly, mainly due to their physical and mechanical properties, such as excellent strength (3.1–4.5 GPa), high modulus of elasticity (220–800 GPa), and low density (1.7–2.1 g/cm^3^) [4,5]. For this reason, different industrial sectors, mainly aerospace and automotive, typically employ CFRPs in a wide number of applications [6]. In numbers, this translated to approximately 140,000 tons of CFRP used in 2020 [7], with use expected to grow more than 12% by 2024. In addition, the volume of the market is expected to grow from USD 4.7 billion in 2019 to USD 13.3 billion by 2029 [8].

However, at the same time, the amount of CFRP waste volume increased significantly, reaching around 4 million tons in 2017 worldwide [9]. This situation highlights the need to manage this waste in a more sustainable way in order to mitigate the environmental impacts. Despite the fact that the European Community has indicated a drastic reduction in the amount of waste to be sent to landfill [10,11], in Italy, there is still some debate about the most appropriate waste management for carbon fibers. The European Waste Catalogue (EWC) classified “wastes from composite materials (impregnated textile, elastomer, plastomer)” with the code 040209.

For this purpose, many efforts have been aimed at recovering and recycling carbon fibers, focusing either on the technology of the resulting properties. A preliminary study carried out by [12] showed that the recycling of CFRP was feasible when working at low temperatures, less than 30 °C, in order to harden the resin and make pre-pregs cuttable, and keeping fixed many of the original properties.

Recycling and re-manufacturing processes are increasing in number and type, as demonstrated in works by Oliveux et al. [13] and Pimenta et al. [14]. In this research, different types of recycling technologies for CFRP are described, including mechanical treatment, pyrolysis, and fluidized bed and chemical treatments, demonstrating the technical feasibility of re-introducing recycled parts into the market for non-critical structural applications. Saccani et al. [15] investigated the possibility of recycling wastes from CFRP, coated in epoxy or another matrix, without any prior chemical or high temperature treatment, to be used to increase the flexural strength and toughness of different mortars. 

Unfortunately, most CFRP wastes are currently landfilled, squandering resources and money, because even though there are several recycling approaches or general recovery techniques available at a laboratory scale, there is scarce possibility to find the same efficiency on a commercial scale. To overcome this issue, other researchers have tried to mix carbon fibers with cementitious or concrete mortar in order to improve their mechanical properties. For example, [16] investigated different solutions referring to the problem of the organic compounds of resins on the surface of fibers, which decrease the bond between the cement and the carbon fibers. Others [17,18,19,20] have focused on the improvement of mechanical properties, such as strength, flexural strength, impact resistance, compressive strength, tensile strength and modulus of elasticity, by mixing carbon fibers with cement in concrete and by testing several samples and specimens. Rangelov et al. [21] considered the possibility of incorporating carbon fiber composite in pervious concrete for pavement applications in order to improve its mechanical properties. Zhang et al. [22] investigated the improvement provided by epoxy-coated CFRP laminates for a beam construction to enhance mechanical properties such as load-carrying capacity, fracture toughness and fracture energy; a configuration that incorporates CFRP laminates could be a potential replacement for steel reinforcement in concrete beams. So far, only a few dedicated approaches have been reported in the literature that consider both the mechanical and environmental performances of CFRP waste in cementitious composites. Recently, Akbar and Liew studied the recycling potential of carbon fiber waste deriving from the aeronautical and wind power sectors; the authors successfully used the recycled fibers for cement composites to improve their mechanical and environmental performance, consequently diverting these wastes from landfill [23]. In addition to these examples, a clear insight into the potential environmental benefits deriving from substituting the mortar constituents (e.g., cement, aggregates, etc.) with CFRP wastes is still desired.

The EU strongly drives all industrial sectors to consider a circular economy, which is an industrial/economic model useful for addressing the enormous environmental problems caused by production processes as well as the lifestyle of the industrialized world. The concept of a circular economy is based on a self-regeneration like that of natural ecosystems, in which materials of biological origin are designed to be reintegrated into the biosphere; using the same principle, technical materials must be designed to be reintegrated into the industrial system, without deep and continuous damage to the biosphere. In this context, the construction sector is undertaking interesting steps, particularly with regard to scientific research [24].

This study proposes a model, in line with circular economy objectives, capable of implementing a feasible industrial symbiosis between two sectors in a win-win scenario. On the one hand, there is the need for industrial companies using CFRP pre-preg sheets to ensure sustainable and inexpensive waste management; on the other, there is the need to move towards more sustainable cementitious products. To this aim, this paper compares the mechanical and environmental performance of an ordinary cement mortar with two types of fiber reinforced mortars; the first type contains a precise amount of carbon fiber (obtained from CFRP waste from an Italian techno-textile company) substituting for sand, whereas the second type substitutes an amount of cement with recycled CFRP fibers. An attributional and comparative LCA (life cycle assessment) [25,26] is carried out on different specimens on a lab scale, which are preliminarily tested and compared in terms of flexural and compressive strength.

## 2. Materials and Methods

**Figure 1:**
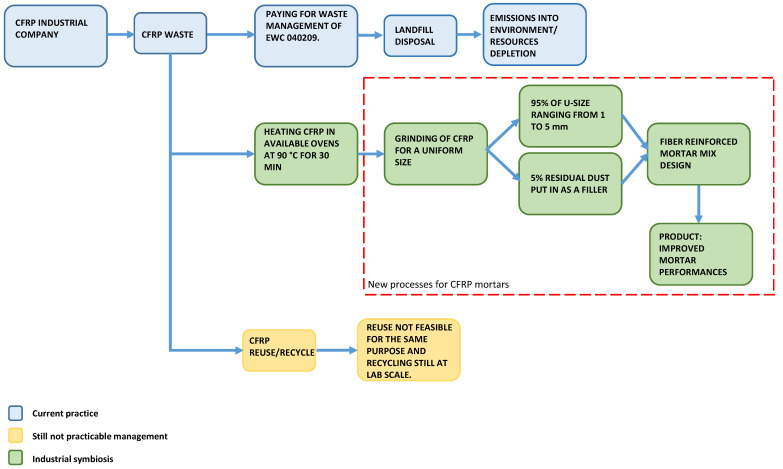
Schematic workflow of the proposed industrial symbiosis.

The model proposed in this work includes different stages, which are summarized in the working path represented in Figure 1.

Figure 1 shows a schematic flow for the post-production process of CFRPs in the context of composites employed in the automotive industry, depicting three different and possible routes of waste management. The first is represented by the light blue color and describes the current waste management process, in which the company pays for a service that transports the waste to a sanitary landfill. Although still operating in Italy, this solution is the least advantageous since it leads to strong economic loss and natural resource depletion.

The second route (the yellow one) refers to the possibility of recycling CFRP waste by reintroducing it in the existing production process. Recycling or reuse should be the most affordable solution in the coming years and can really add value to this material.

The “green” route describes the industrial symbiosis approach suggested within the framework of this study. The first step considers the heating of the CFRP pre-preg waste (usually different pieces of small size, not suitable for other applications) in the oven of the production plant in order to harden the epoxy resin of the coating and to remove the sticky properties. This process still takes place in the company’s furnace, benefiting from the firing of CFRP profiles that must be molded for the company’s appropriate commercial purposes. This step does not produce any significant increase in time and temperature; for this reason, in Figure 1, the heating phase remains outside of the dashed line that delimitates the new process.

Based on the proposed waste management flow, an experimental campaign was conducted in the laboratory of the Department of Structures for Engineering and Architecture, University of Naples “Federico II”, aiming to optimize the mechanical performance of cementitious material reinforced with short carbon fibers deriving from CFRP waste from the automotive industry. In order to study the effects of short carbon fibers on the mechanical behavior of the CFRP cementitious composites, flexural and compression tests were carried out on different groups of mortars with and without carbon fibers.

### 2.1. Carbon Fibers

The CFRP material used in this study is supplied by an industrial company (Adler Pelzer Group, Airola, Italy) in the form scraps/waste of “pre-preg”, i.e., carbon fiber sheets pre-impregnated with a pre-catalyzed resin system. Table 1 shows the properties of carbon fibers used for conducting the experimental process, provided by the manufacturer and determined according to EN ISO 527-4 [27].

### 2.2. CFRP Cementitious Composites: Production Route and Mixing Process

**Figure 2:**
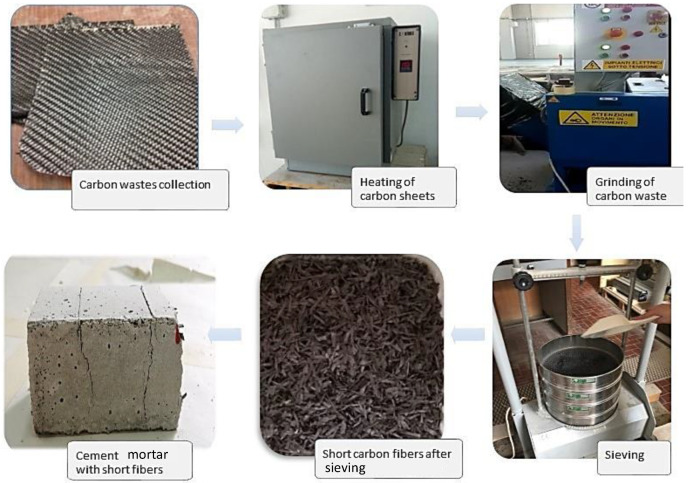
Recycling process and fiber reinforced mortar production.

As mentioned above, the CFRP recycling process (green flow in Figure 1) aims to produce a better performing mortar in terms of mechanical properties, meaning an increased value of compression/tensile strength. Indeed, the addition of carbon fibers in the mortar potentially leads to (i) a reduction of micro-cracks caused by plastic shrinkage due to evaporation of the water into the mix, and (ii) a bridging effect over the crack itself. Hence, a production process for CFRP cementitious composites was developed, consisting of the steps summarized in Figure 2.

Figure 2 shows the recycling process in more detail and with some illustrations. The first step is the collection of pre-preg scraps/waste from industry storage. These items are no longer useful for the purpose for which they were designed, i.e., automotive. Then, in order to eliminate the sticky coating resulting from the resin pre-impregnation (which makes these fibers difficult to process), these scraps are heated in an oven at a temperature of about 90 °C for 30 min. This step allows the polymerization of the pre-preg sheets, resulting in a hardened product. This process takes place in the company’s autoclave, benefiting from the curing of CFRP profiles that are manufactured for the company’s appropriate commercial purposes. This is an important aspect because it avoids adding energy burdens to the recycling process for CFRP waste.

After heating, the stabilized CFRP wastes are ready to be chopped up into suitably smaller fragments that can be easily worked into mortars. In fact, the third illustration of Figure 2, refers to the grinding of CFRP heated pre-pregs, a pre-treatment operation consisting of reducing the size of the material into fragments using a mechanical grinder. Afterwards, the fibers are of different sizes, and therefore it is necessary to use a sieve in order to select fibers of the desired size range. Thus, by using a series of vertical sieves, with the opening gradually decreasing from top to bottom, the separation of grain sizes according to the selected openings is obtained. This procedure allows for the identification of the length of fiber fragments in order to define optimal CFRP mortar mixes. One of the most sensitive aspects is related to the production of a residual dust (usually around 4%) during the grinding process; nevertheless, this residual component could be introduced into the mortar as a filler. Once the fibers of the desired size have been selected, they are dry-mixed with the sand and cement; after a homogeneous dry mixture is obtained, the necessary water is added, and it is mixed again. Afterwards, the mortar is ready to be cast into molds to obtain test specimens for measurement of the mechanical properties.

### 2.3. Dimensional Distribution

**Figure 3:**
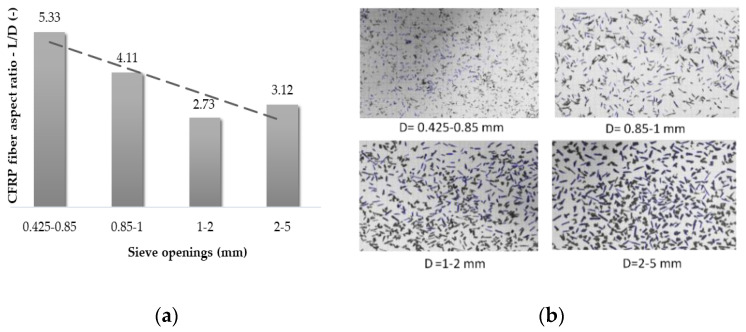
(**a**) Aspect ratio distribution as a function of sieve openings; (**b**) fiber tracking as the dimensions of sieve opening change.

By means of a MATLAB routine (FiberApp) it was possible to report information in terms of CFRP fiber length and diameter statistical distribution (Table 2). In particular, this application allowed the processing of fiber data directly from the pictures of randomly distributed fibers placed on a flat surface after the sieving operation, as shown in Figure 3b. The statistical results are reported in terms of mean value of fiber length and fiber diameter (with the corresponding standard deviation, σ) for each range of sieve opening. It is worth noting that the mean value of the fibers’ length is larger than the dimensional range of the corresponding sieve diameter due to the oblong shape of the fibers (smaller diameter than length), which facilitates the insertion into tighter openings during the separation process. An important result was the determination of the aspect ratio of the fibers after the sieving process, i.e., the ratio between the expected value of the fiber length and diameter (shown in Table 2), which is reported by means of the histogram in Figure 3a. As the aspect ratio increases, the bonding surface between the CFRP and mortar material increases, and consequently the performance of the fiber mixture improves since the fibers subjected to tensile forces are more difficult to slip off.

### 2.4. Fiber Reinforced Mortar Samples for Mechanical Testing

In this study, an ordinary cementitious mortar was used as a reference base material for the comparison with fiber reinforced mortars. The reference cement mortar was obtained starting from a R32.5 strength class and using the constituents and preparation procedure specified in UNI EN 196-1 [28]. Then, the experimental assessment approach followed different criteria for the fiber reinforcement specimens’ production: (a) workability; (b) fiber volume fraction (expressed in percentage with respect to the total volume of the mixture); (c) fiber length; (d) substitution of mortar components (cement and/or sand). In criterion (d), a pre-defined volume of cement or sand was substituted with the same volume of recycled CFRP fibers. Therefore, the different CFRP mortar mixes are herein identified with the volume fraction of recycled fibers in relation to the total volume of the mixture. For a given interval of fiber length distribution deriving from the sieving process (i.e., first column of Table 2), the maximum fiber content (or, equivalently, the maximum volume fraction) was determined considering the workability of the fresh mortar. This requirement was set using a mini-conical slump flow test in order to guarantee an effective pouring of the fiber reinforced mortars into the molds.

Mix proportions of each CFRP mortar composite series are given in Table 3 and Table 4, where the label SS or SC refers to mix design criterion (d), i.e., taking into account the sand (S) or cement (C) substitution, respectively. R32.5 is the strength class of the cement adopted, according to UNI EN 197-1 [28]. The percentage refers to the amount of recycled CFRP fibers (volume fraction) in the sample, and the subscript intervals stand for sieve size.

With regard to the preparation procedure, the amounts of sand, cement, and recycled CFRP fibers were preliminarily dry mixed manually in order to ensure a uniform distribution of fibers in the powders. The amount of water was carefully added to the dry mix, and afterwards the resulting components were mixed thoroughly for 4 min with a mixer. Then, the fresh mortar was poured into 160 mm × 40 mm × 40 mm molds and cured, after demolding, for 28 days.

The CFRP composite mortar specimens were tested under flexural and compression loading conditions, according to UNI EN 196-1: 2016 [29] protocol. Specifically, the flexural strength of the mortar was determined by means of three-point bending tests carried out on prismatic specimens (160 mm × 40 mm × 40 mm) up to failure, while the compressive strength was determined on the two halves resulting from the flexural test. For each specimen series, three samples were tested under flexural conditions and, consequently, six samples under compression load. In each specimen series, the samples were tested using a servo-hydraulic universal machine with load capacity of 200 kN under displacement control. The resulting average mechanical characteristics are reported and discussed in the Results section.

### 2.5. Life Cycle Assessment

#### 2.5.1. Goal and Scope Definition

##### The System under Analysis and System Boundaries

As mentioned above, the aim of the study is to quantify the mechanical and environmental performances of an ordinary cement mortar compared with two experimental mortars. The study also takes into account the potentially avoided impacts as a result of this industrial symbiosis, in particular those related to the prevention of the landfilling of CFRP, which in Italy remains the typical way to manage this kind of waste.

An attributional and comparative LCA (life cycle assessment) in agreement with the ISO standard [25,26] was carried out for different specimens on a lab scale.

The activity was based on the analysis of several factors: the quantity and size of the fibers, the water/cement ratio, and the way in which the fibers are incorporated. The system analyzed refers to cement mortars produced in the laboratory in accordance with UNI EN 196-1 [29], which indicates the proportions between the components of the standard mortar and water/concrete ratio. The system includes specimens of the same size, compared at the same environmental conditions, and submitted to the same experimental tests. In particular, an ordinary cement mortar was compared with two experimental mortars: in the first one, a precise amount of carbon fibers substitutes for a share of sand, and in the second one, fibers substitute for a share of cement.

The functional unit corresponds to each single specimen, having size of 40 mm × 40 mm × 160 mm and being realized in the laboratory and suitable for the tests. It includes the different quantities of cement, water, sand, and fibers in each specimen.

##### Data Quality

The LCA study was carried out by means of SimaPro 8.0.5, a dedicated tool provided by Pré-Consultant [30] and recommended for this kind of analysis. The software is able to calculate along the different stages of LCA the impacts of the product/service/good to analyze; it includes different databases embodying all the environmental data and other information related the production processes or elaborations for the selected object. The data derive from the Ecoinvent v.3.1 database [31], one of the most reliable databases on the market, with thousands of items. In particular, the production processes referring to cement, sand, and water have been chosen and modified according to research needs, in order to reduce the range of uncertainties [32,33,34,35] and make them as close as possible to the real case.

##### Life Cycle Impact Assessment—LCIA—Methodology

LCIA—life cycle impact assessment—is the stage of an LCA during which all the environmental burdens (different emissions to air, water, soil etc.) previously collected in the LCI—life cycle inventory—are transformed in terms of results, such as into environmental indicators that are much more comprehensible, even to a non-specialist audience. This stage requires the use of a mathematical model to convert values for all types of emissions, energy, heat dissipation, noise, etc., into impact categories to provide essential information for a decision-making process. IMPACT 2002+, the methodology used for this study [36], is a recognized tool previously used in other studies [37,38]. It transposes the LCI results into 15 midpoint categories; after this it is possible, by reducing the complexity and at same time losing scientific information, to transform the data in four damage categories more suitable for a non-scientific public. The presentation of the final LCA results can be limited to a few impact categories, chosen as the most representative for the environmental burdens, to summarize the scenario. In this case, six impact categories were selected: respiratory inorganics (RI), terrestrial ecotoxicity (TE), land occupation (LO), global warming (GW), non-renewable energy (NRE), and mineral extraction (ME).

#### 2.5.2. Life Cycle Inventory—LCI

The life cycle inventory (LCI) in this study is based on the quantity of material to be produced and the energy necessary to grind the CFRP fibers. The amount defined for the experimental test, in terms of water, cement, and sand is suggested by [29], and the values are reported in Table 4, together with the amount of CFRP used to substitute sand in the first case (upper part) and cement in the second one (lower part).

Furthermore, the system considers also the amount of electricity needed for the grinding machine to form regular sizes of CFRP. A technical data sheet was used to calculate the energy consumption of the machinery. The internal size of the machinery is 0.6 m × 0.4 m × 0.25 m, and thus the internal volume is 0.06 m^3^. It was assumed that it regularly works with a load of CFRP around 10% of the total volume; thus, the total working volume is 0.006 m^3^ with a total weight of 0.8–0.9 kg. The amount of carbon fiber necessary is for a specimen range of 36 g to 72 g; thus, the energy accountable to the milled CFRP needed for the mixture of a sample is around 5% of the share of the total energy consumption.

The grinding machine has a nominal power of roughly 400 W; thus, the time to grind a total load (0.8–0.9 kg) is 30 min and the supposed time needed only for the fibers necessary for the quantity of one specimen is less than 2 min. Therefore, the energy consumption is less than 0.01 kWh.

The life cycle inventories for cement, sand, and water come from the database Ecoinvent 3.1; the items selected are “Cement, Portland production” for cement, “Silica sand” for the amount of sand, and “Tap water production” for the use of water. All production sites are located in the Campania region, south of Italy, and all processes are similar to those selected from the database. In this case, it is important to declare that the process data sheets for cement and sand were modified for transportation items, deleting all entries related the globalized market, which include transportation among different countries, not only considering road freight but also considering sea freight. Another pivotal modification concerns the electricity mix for the production of cement and sand and the distribution of water. In particular the Italian energy mix was chosen based on the most recent data available [39].

A sanitary landfill was chosen in order to take into account the burdens related to the baseline scenario, where carbon fibers are sent to landfill, and no recycling or reuse has been adopted. As indicated also by [7], the LCI refers to mixed plastic landfill, based on the study of Doka [40], which provides information necessary for the study.

## 3. Results

### 3.1. Mechanical Performance

The CFRP composite mortar specimens were tested under flexural and compression loading conditions according to UNI EN 196-1: 2016 [29] specifications. With regard to the different mixes, the results are summarized in Table 5 and Table 6, which report the outcomes in terms of average flexural and compression properties, respectively, along with the results of the statistical analysis for each specimen series.

With reference to the samples in substitution of sand, the analysis of the results showed a generalized increase in flexural and compressive strength compared to the reference sample (without fibers). Specifically, flexural and compressive strength increases up to 18% and 11%, respectively, were recorded for the specimens with a volume fraction of CFRP fibers of 4% and with average lengths ranging between approximately 4.40–8.70 mm (i.e., obtained from sieving openings of 1–2 and 2–5 mm). On the contrary, smaller fiber lengths were not able to provide a satisfactory load transfer between matrix and reinforcement, resulting in limited or negligible strength increases (e.g., SS-R32.5_4%_0.425–0.85 series). In the case of CFRP reinforced samples obtained in substitution of cement, a satisfactory response was exhibited only in the case of flexural loading, whereas the compression behavior was unchanged or even worsened. In detail, a larger substitution of cement with CFRP waste fibers (i.e., in the case of SC-32.5_12.8%_2–5 series) generated a detrimental effect, probably due to voids and imperfections between matrix and fibers, that led to a decrease of strength up to 30% with respect to the non-reinforced counterpart. Among the cement substitution cases, the SC-R32.5_6.4%_2–5 series exhibited the best mechanical performance. In this case, the CFRP mortar was characterized by a limited increase of both flexural and compressive strength; however, globally, the performance was at least similar to (not worse than) the reference mortar material but was obtained using a reduced amount of cement, i.e., achieving a more sustainable product. In other words, the lower binder content was balanced by the reinforcing fibers obtained from CFRP wastes. In fact, this optimal result, combined with a circular economy approach and by means of the application of industrial symbiosis principles, enables the possibility of reducing environmental impacts as a result of reduced cement use.

**Figure 4:**
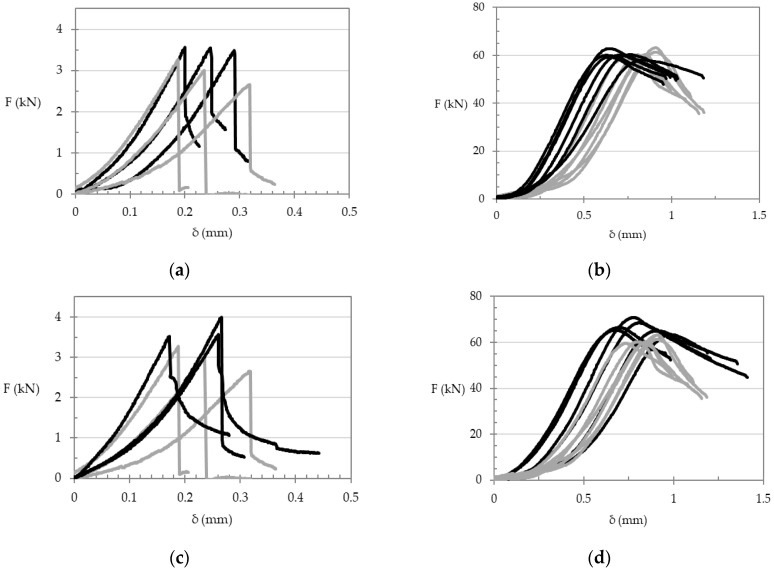
(**a**) Load-deflection relationship in flexural regime (grey lines: control specimens, black lines: SC-R32.5_6.4%_2–5 series); (**b**) load-deflection relationship in compression regime (grey lines: control specimens, black lines: SC-R32.5_6.4%_2–5 series); (**c**) load-deflection relationship in flexural regime (grey lines: control specimens, black lines: SS-R32.5_4%_2–5 series); (**d**) load-deflection relationship in compression regime (grey lines: control specimens, black lines: SS-R32.5_4%_2–5 series).

**Figure 5:**
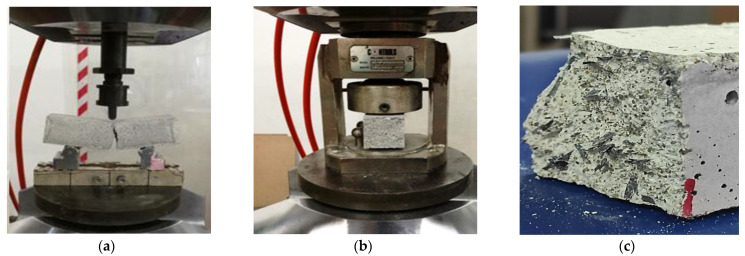
(**a**) Example of flexural test at failure; (**b**) example of compression test executed on one half of the broken flexural specimen; (**c**) fractured cross-section of the SS-R32.5_4%_2–5 series specimen highlighting the fiber distribution within the cementitious matrix.

Four representative load-deflection curves of the flexural and compressive tests carried out on the CFRP reinforced mortars are illustrated in Figure 4 and compared to the control specimens without fibers. Referring to the stiffness properties, a more significant effect was recorded for the flexural specimens. A qualitative comparison of the stiffness properties was made on the basis of the slopes of the flexural load-deflection curves in the range of 1/3–2/3 of the peak load; this interval was chosen to avoid the initial part of the diagrams, which were characterized by a nonlinear tendency related to the contact between the specimen and the loading device (i.e., contact law). In detail, the SC-R32.5_6.4%_2–5 series and SS-R32.5_4%_2–5 series exhibited a more stiff response than the reference series (without fibers), as demonstrated in Figure 4a,c. In particular, the flexural stiffness demonstrated by the specimens characterized by fibers in substitution of sand in 4% by volume of mixture was equal to 14.07 kN/mm; whereas the specimens characterized by fibers in substitution of cement in 6.4% by volume of mixture had an average stiffness of 13.83 kN/mm. In both cases, an increase of more than 20% was seen in comparison with the specimens without fibers (i.e., equal to 11.07 kN/mm), due to the reinforcing effect provided by the CFRP waste fibers. Figure 5 includes sample pictures of the test results discussed above.

### 3.2. Environmental Performance

**Figure 6:**
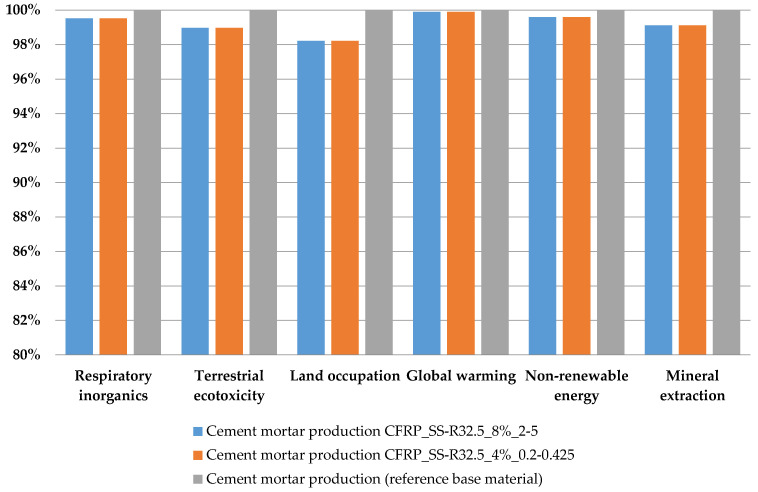
Characterization of environmental impacts for three different mortar mixtures.

**Figure 7:**
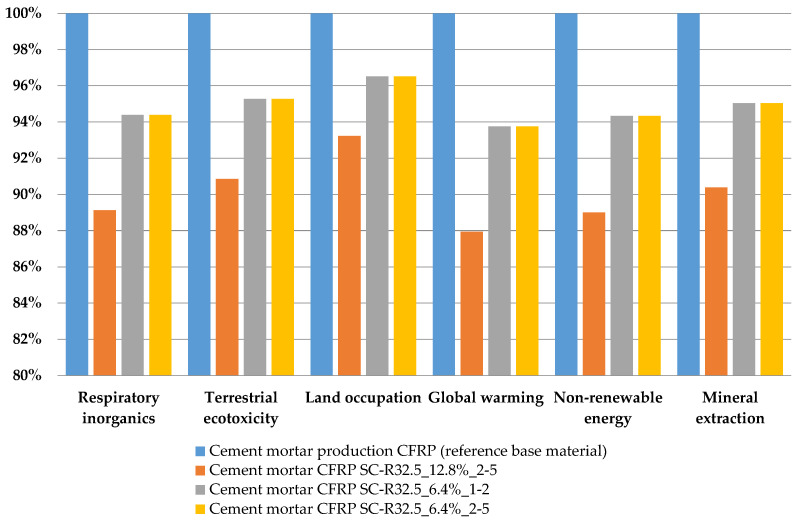
Characterization of environmental impacts for four different mortar mixtures.

**Figure 8:**
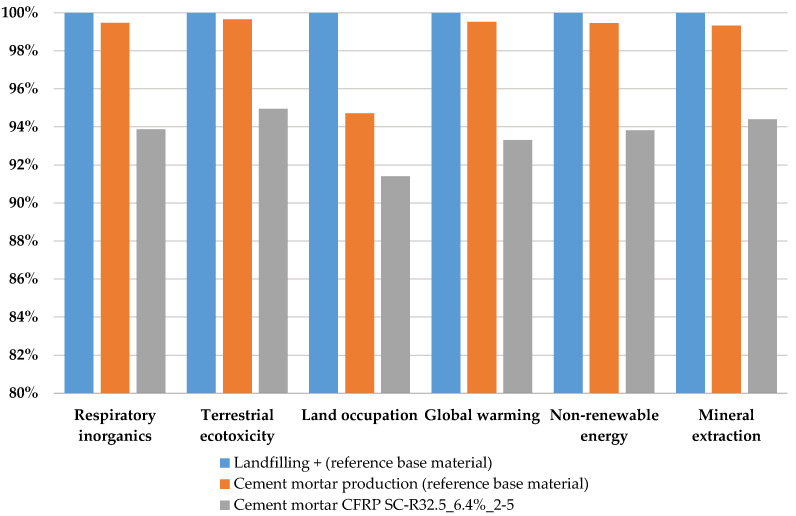
Characterization of environmental impacts considering also landfilling as waste treatment for carbon fiber.

Based on the data from the LCI, it was possible to calculate the LCIA. As mentioned above, the impact categories selected are respiratory inorganics (RI), terrestrial ecotoxicity (TE), land occupation (LO), global warming (GW), non-renewable energy (NRE), and mineral extraction (ME). Figure 6, Figure 7 and Figure 8 are expressed in terms of percentage for each impact category (from 0 to 100, where 100% is the specimen with the higher impact); however, the vertical axes start from 80% or 90% in order to facilitate readability and better highlight the differences between the results of the different mortars.

Figure 6 refers to the characterization of the first set of specimens, which encompasses only the substitution of sand. These results report the avoided environmental burden related to the amount of sand substituted with the CFRP fibers, and particularly refer to the avoided extraction of raw materials. The modified mortars with the amount of 4% and 8% of CFRP show a minimum and insubstantial decrease in terms of impact with reference to the baseline mortar. In fact, for the six categories, there are no significant reductions of environmental impact accountable to “SS-R32.5_4%_0.2–0.425” and “SS-R32.5_8%_2–5”, which, at the most, reach 3% for the LO category. This shows that the use of CFRP fibers in substitution for sand in the mixture is able to provide only moderate environmental benefits. 

Figure 7 shows the results of the characterization comparing the baseline cement mortar and mortars with a substitution of cement. There are substantial environmental benefits depending on the amount of replaced cement. In fact, mortar with a substitution rate of 6.4% when compared to the baseline mortar present environmental benefits around 5%, while mortar with a substitution rate of 12.8% shows benefits of around 10% along the six categories.

The last part (Figure 8) of this assessment takes into account the environmental impacts related to the landfilling of carbon fiber, which is still the waste treatment adopted in large parts of Europe and Italy. The graph depicts the LCIA results of the characterization, where the environmental impacts of landfilling—36 g of CFRP waste, which is the minimum amount substituted—are added to those of the baseline mortar. These two shares were merged (blue bars) in order to define the environmental burdens concerning the actual conditions, identified as the payment by the techno-textile company for sending the waste to landfill. The histogram then compares the environmental impacts with those of the baseline mortar (without any substitution, the orange bars) and those related to the SC-R32.5_6.4%_2–5 mortar (grey bars). The latter presents a significant environmental benefit, varying from 5% to 10%, for all the midpoint categories selected.

## 4. Discussion

The combination of experimental and LCA results revealed that the substitution of cementitious mortar constituents (either binder or aggregates) with recycled short carbon fibers has the potential to provide interesting properties concerning both the mechanical and environmental aspects. Referring to the flexural and compression performances, summarized in the Table 5 and Table 6, the reinforced mortars obtained with the substitution of sand demonstrated a satisfactory improvement in the case of fiber content of 4% (by volume of mixture) and fiber lengths of approximately 8 mm (i.e., obtained with 2–5 mm sieving openings). This mix showed an increase, with respect to the reference material, of the flexural and compression strengths of 18% and 10%, respectively, as well as a significant increase in the flexural stiffness, quantified as 27%.

Concerning the mixes with the substitution of cement, the improvements were less significant; the optimal result was achieved with the amount of CFRP fibers of 6.4% by volume of mixture and same optimal length of the sand substitution case, i.e., approximately 8 mm (obtained with 2–5 mm sieving openings). Even though the flexural behavior was improved, the reduction of cement had a detrimental effect in compression; this circumstance was expected due to a lower binder content. The CFRP reinforced samples did not worsen the compressive strengths with respect to the reference mortar produced with a higher cement content. With regard to the observed discrepancy between flexural/compression improvements, it is worth noting that our results are in line with the majority of the existing experimental studies available in the literature dealing with fiber reinforced mortars/concretes. It has been demonstrated that the compressive strength is less influenced by the increase of the fiber content, whereas the tensile behavior and especially the post-cracking and residual strength is greatly improved [41].

In the mixes (SS or CS) obtained with optimal fiber lengths—i.e., approximately 8 mm and obtained with 2–5 mm sieving openings—the mechanical improvements were probably associated with the fiber aspect ratio, which allowed an effective distribution of fibers around the cementitious matrix. In this way, some additional energy was necessary to de-bond the CFRP fibers and then pull them out, resulting in larger fracture energies of the fiber reinforced specimens. Furthermore, failure of the CFRP fibers in the optimal mixes reasonably involved rupture, preceded by some de-bonding. Reducing the optimal lengths and/or adding much more CFRP fibers resulted in a poorer fiber dispersion, inefficient fiber orientation, and the formation of small air pockets. If the fiber concentration was too high or too low, or if the fibers were not aligned in the direction of the applied stress, the corresponding energy absorbing efficiency was not optimized. These results also suggest that the optimal CFRP fiber lengths could provide crack control due to the tensile stress transfer capability of the fibers across crack surfaces (i.e., crack bridging). However, more experiments involving larger specimens will be needed to further investigate a possible pseudo-ductile tensile response of the optimal mixes as well as the enhanced energy dissipation capabilities [42].

**Figure 9:**
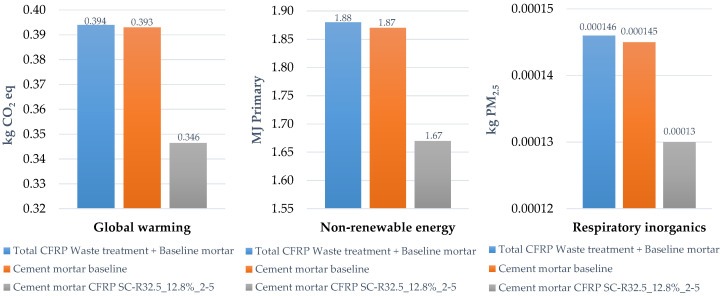
Comparison of environmental impacts by considering absolute values for three impact categories and for three cement mortars.

Finally, considering the SC-R32.5_6.4%_2–5 sample as one of the optimal solutions, on the one hand a good mechanical optimization is guaranteed, and on the other hand a sustainable perspective is reproduced, as demonstrated by the life cycle assessment. In fact, the results of the life cycle assessment support those of the experimental tests for mechanical performance. Specifically, as observed in Figure 6 and Figure 7, the replacement of sand by reduced content does not disclose great benefits from an environmental point of view. In contrast, when specimens replacing cement were analyzed, the benefits were higher, especially for the SC-R32.5_12.8%_2–5 series, with savings of more than 10% for most of midpoint categories analyzed. In fact, as depicted in Figure 9, for GW, from 0.394 kg of CO_2_ of the baseline to 0.346 kg CO_2_ (around 12%) of the experimental mix for NRE, a comparable improvement is recorded (around 11%) from about 1.88 MJ primary of the baseline to about 1.67 MJ primary of the experimental mix; the same gain (11%) is reported for RI, from 0.000146 kg of PM_2.5_ to about 0.00013 of the experimental mix. Furthermore, the results highlight the necessity of an appropriate waste treatment for carbon fiber, especially in countries like Italy, where there is not enough space to place landfills, making these the least advantageous waste management option as suggested by (Directive 98/2008 EC). In fact, disposal by landfill can result in big losses of resources in terms of land, space, and mineral natural extraction; it also means losing the business opportunities offered by industrial symbiosis, as encouraged in a circular economy perspective.

## 5. Conclusions

The aim of this paper was to quantify the environmental and mechanical performance of an ordinary cement mortar compared with two mortars containing carbon fiber waste/scraps. Through a circular economy application, the traditional (and linear) production model based on the use of natural resources and oriented only to the maximization of mechanical performance was modified following an industrial symbiosis model. With this kind of approach, any production process has to be designed in order to replicate as much as possible the “circularity” of material flows, typical of natural systems. To this purpose, this work developed an integrative criterion of recycling short carbon fibers as derived from CFRP from the automotive industry and reused within the construction industry, especially for cement mortar production. The results of this work showed that mortars obtained with the substitution of a certain amount of cement or sand with short carbon fibers from CFRP scraps had the potential to increase mechanical performance when compared to ordinary cement mortar, especially in terms of flexural strength and ductility. Furthermore, an attributional and comparative life cycle assessment demonstrated how sustainable this process could be from an environmental point of view by means of resource optimization and diverting waste from landfill. This process could become a tool for embracing different critical aspects related to the built environment and leading to sustainable development paths. After the preliminary assessment herein reported and starting from the optimal mixes exhibiting the best mechanical/environmental performance, further developments include (i) a better characterization of the fibers’ treatment and selection in association with microstructural analyses to assess the micro-level interactions; (ii) the characterization of the fracture energy capability deriving from the use of CFRP waste fibers; (iii) enlarging the boundary system of the LCA.

## Figures and Tables

**Table 1:** Properties of CFRP pre-pregs.

Tensile Strength	Elastic Modulus	Density
1061.9 MPa	200–600 GPa	1750 Kg/m^3^

**Table 2:** Dimensional distribution results.

Sieve Openings (mm)	Length		Diameter		L/D (-)
	Mean Value (mm)	StDev-σ (mm)	Mean Value (mm)	StDev-σ (mm)	
0.425–0.85	2.84	0.30	0.53	0.39	5.33
0.85–1	3.77	0.29	0.92	0.38	4.11
1–2	4.40	0.28	1.61	0.47	2.73
2–5	8.72	0.32	2.79	0.34	3.12

**Table 3:** Properties of mortar specimens in substitution of sand and cement.

Specimen ID	Reference Mortar (UNI EN 196-1: 2016 [29])	Amount of Fibers—Volume Fraction (%)	Mean Fibers Length (mm)	Sieve Openings (mm)
Sand substitution				
S-R32.5	standard	-	-	-
SS-R32.5_4%_0.425–0.85	standard	4	2.84	0.425–0.85
SS-R32.5_4%_0.85–1	standard	4	3.77	0.85–1
SS-R32.5_4%_1–2	standard	4	4.40	1–2
SS-R32.5_4%_2–5	standard	4	8.72	2–5
SS-R32.5_8%_2–5	standard	8	8.72	2–5
Cement substitution				
S-R32.5	standard	-	-	-
SC-R32.5_6.4%_1–2	standard	6.4	4.40	1–2
SC-R32.5_6.4%_2–5	standard	6.4	8.72	2–5
SC-32.5_12.8%_2–5	standard	12.8	8.72	2–5

**Table 4:** Series of sampling with substitution of sand (upper part) and cement (lower part).

Specimen ID	Cement (g)	Water (g)	W/C (-)	Sand (g)	Fibers (g)	Volume Fraction Substituted with CFRP Fibers (%)
Sand substitution						
S-R32.5	450	225	0.5	1350	-	-
SS-R32.5_4%_0.425–0.85	450	225	0.5	1296	36	4
SS-R32.5_4%_1–2	450	225	0.5	1296	36	4
SS-R32.5_4%_0.85–1	450	225	0.5	1296	36	4
SS-R32.5_4%_2–5	450	225	0.5	1296	36	4
SS-R32.5_8%_2–5	450	225	0.5	1242	72	8
Cement substitution						
S-R32.5	450	225	0.5	1350	-	-
SC-R32.5_6.4%_1–2	421.2	225	0.53	1350	36	6.4
SC-R32.5_6.4%_2–5	421.2	225	0.53	1350	36	6.4
SC-R32.5_12.8%_2–5	394.4	225	0.57	1350	72	12.8

**Table 5:** Flexural response of composites with various fiber lengths and content.

Specimen ID	Average Flexural Strength Reference Mortar (MPa)	Average Flexural Strength CFRP Reinforced Mortar (MPa)	DevStd Flexural Strength (MPa)	Coefficient of Variation (%)	Variation of Flexural Strength Δ (%)
Sand substitution					
S-R32.5	7.53	-	0.26	3.5	-
SS-R32.5_4%_0.425–0.85	-	7.38	0.91	12.3	−2
SS-R32.5_4%_0.85–1	-	8.65	0.24	2.8	+15
SS-R32.5_4%_1–2	-	7.94	0.31	3.9	+5
SS-R32.5_4%_2–5	-	8.88	0.66	7.4	+18
SS-R32.5_8%_2–5	-	8.68	1.34	15.4	+15
Cement substitution					
S-R32.5	7.53	-	0.26	3.5	-
SC-R32.5_6.4%_1–2	-	8.45	0.33	3.9	+12
SC-R32.5_6.4%_2–5	-	8.36	0.08	1.0	+11
SC-R32.5_12.8%_2–5	-	6.47	0.1	1.5	−14

**Table 6:** Compression response of composites with various fiber lengths and content.

Specimen ID	Average Compressive Strength Reference Mortar (MPa)	Average Compressive Strength CFRP Reinforced Mortar (MPa)	DevStd Compressive Strength (MPa)	Coefficient of Variation (%)	Variation of Compressive Strength Δ (%)
Sand substitution					
S-R32.5	38.01	-	0.87	2.3	-
SS-R32.5_4%_0.425–0.85	-	40.41	1.93	4.8	+6
SS-R32.5_4%_0.85–1	-	42.34	1.65	3.9	+11
SS-R32.5_4%_1–2	-	39.62	1.48	3.7	+4
SS-R32.5_4%_2–5	-	41.72	1.67	4.0	+10
SS-R32.5_8%_2–5	-	38.55	1.66	4.3	+1
Cement substitution					
S-R32.5	38.01	-	0.87	2.3	-
SC-R32.5_6.4%_1–2	-	35.76	2.59	7.2	−6%
SC-R32.5_6.4%_2–5	-	37.68	0.97	2.6	−1%
SC-32.5_12.8%_2–5	-	27.76	2.91	10.5	−27%

## Data Availability

The data presented in this study are available on request from the corresponding author.
